# A Measure of Suffering in relation to Anxiety and Quality of Life in IBS Patients: Preliminary Results

**DOI:** 10.1155/2017/2387681

**Published:** 2017-07-04

**Authors:** Sanda Pletikosić Tončić, Mladenka Tkalčić

**Affiliations:** Department of Psychology, Faculty of Humanities and Social Sciences, University of Rijeka, Sveucilisna Avenija 4, 51000 Rijeka, Croatia

## Abstract

Irritable bowel syndrome (IBS) is a chronic gastrointestinal (GI) disorder with a severe impact on quality of life (QoL). We explored the relationship of a visual measure of suffering, the PRISM-RII, with quality of life (QoL) and anxiety measures in IBS patients. Participants were 44 IBS patients who completed several questionnaires and kept a symptom diary for two weeks. The measures used were PRISM-RII (self-illness separation (SIS); illness perception measure (IPM)); IBS-36 (IBS health related QoL); SF-36 (physical and mental health related QoL); State-Trait Anxiety Inventory (STAI-T); Visceral Sensitivity Index (VSI; GI-specific anxiety); and a symptom diary. SIS was negatively correlated to VSI, while IPM was negatively correlated to SIS and the physical component of SF-36 and positively to VSI and symptom severity. We found significant differences between participants who perceive their illness as small and those who perceive it as medium in SIS, symptom severity, VSI, and the mental component of SF-36. Participants, who perceived their illness as small, represented their illness as more distant, showed lower average symptom severity, and had lower GI-specific anxiety and higher QoL. The results indicate that IPM and SIS can be useful in discriminating patients with more prominent psychological difficulties and QoL impairment.

## 1. Introduction

Irritable bowel syndrome (IBS) is a chronic gastrointestinal disorder, affecting around 10% of the population globally [1]. IBS is characterized by changes in stool form and/or frequency, accompanied by pain and/or discomfort. Functional disorders, such as IBS, are diagnosed based on patient reports about their symptoms, with the help of clear diagnostic criteria, known as the Rome criteria [2]. The Rome criteria categorise IBS patients into subtypes, based on their predominant bowel habits, which is extremely useful for clinical practice and adequate symptom relief for the patient. Most IBS patients, however, are prone to variations in symptoms over time, which consequently means that many of them change subtypes [3]. Other than in predominating symptoms, IBS patients vary greatly in the severity of symptoms which they experience, as well as in the temporal patterns of their symptom flare-ups. Some patients report everyday symptoms while others report episodic flare-ups weeks or months apart. Some describe their symptoms as mild to moderate, while others find them completely debilitating [1, 3]. Consequently, identifying patients with severe symptoms and/or patients with severe quality of life (QoL) impairment is at least of equal importance for overall treatment outcomes as identifying their IBS subtype. The biopsychosocial (BPS) model postulates that IBS is the result of an interaction of complex biological and psychosocial factors, which play predisposing (e.g., genetics, early trauma, and trait neuroticism), precipitating, and perpetuating roles (e.g., gastrointestinal (GI) infection, stressful events, depression, and anxiety) thus contributing to illness onset and symptom maintenance [4, 5].

QoL assessment in IBS patients is usually performed through the use of questionnaires, either for general health related QoL or for IBS-specific health related QoL. Research consistently shows that IBS patients have significantly lower QoL scores compared to healthy controls [6–8] and that their QoL scores on illness-specific measures are indicative of impaired QoL [6, 9]. Compared to inflammatory bowel disease (IBD), IBS presents with symptoms which appear less severe; however most research findings indicate similar levels of QoL impairment in patients with IBS and IBD [10, 11].

In IBS patients, QoL is related to measures of physical impairment such as the functional bowel disorder severity index (FBDSI) and bowel disease questionnaire (BDQ) [12, 13] and measures of psychological dysfunction, such as anxiety and depression [7, 11, 14]. Generally, research shows increased levels of anxiety in IBS patients compared to healthy controls [14–16]. Some IBS patients have psychiatric comorbidities and suffer from anxiety-spectrum disorders; however most of them have heightened but subclinical levels of anxiety-related states [17, 18].

Gastrointestinal-specific anxiety is one such state, which potentially plays a significant role in IBS symptom perpetuation, especially in those patients who are not suffering from anxiety-spectrum disorders. GI-specific anxiety refers to cognitions, emotions, and behaviours related to the digestive system, which are a result of fear and anxiety about GI sensations, symptoms, and the context in which these sensations and symptoms appear [19]. There are findings indicating that GI-specific anxiety is a good predictor of IBS diagnostic status [20], symptom severity [21], and mental QoL in IBS patients [22]. Also, it appears that GI-specific anxiety might be a mediator between trait anxiety and symptom severity [19, 23].

Overall, it seems well established that IBS patients have significantly impaired QoL and show higher levels of psychological distress, specifically of anxiety-related states. The chronic nature of IBS, whose symptoms are often burdening for everyday functioning, indicates that most IBS patients experience substantial levels of suffering. According to Cassell [24], suffering is defined as “the state of severe distress associated with events that threaten the intactness of the person” [pp. 640]. Suffering is experienced when a person perceives an event as threatening and appraises their own coping resources as insufficient. In a similar manner, Chapman and Gavrin [25] define suffering as a result of perceived damage to the integrity of the self—a person's subjective sense of identity. Their definition of suffering also includes a negative cognitive and affective state resulting from a perceived threat and the lack of resources for coping with the threat. In the past, some authors have defined suffering in the context of studying chronic pain [26], viewing suffering as a component of the affective dimension of pain [27]. In recent years, however, researchers have mainly accepted a broader conceptualization of suffering—one that has many possible causes. Although suffering can be related to pain, not all pain causes suffering, nor is all suffering caused by pain [25]. In line with that observation, the same can be said about illness severity in patients with chronic illnesses—suffering is not simply determined by the severity of an illness, rather it is the result of the perceived threat it poses to one's self [28]. In patients with chronic illnesses, there is a disparity between their self-expectations and their actual performance, reflecting the disability caused by various aspects of their illness. This inability to reach their own expectations is what represents a threat to their self-integrity [25]. Since pain is one of the main symptoms of IBS, there are many studies researching the relationship between GI pain symptoms and psychological characteristics of IBS [7, 29, 30]; however there are only a few studies dealing with suffering in IBS patients. In those studies, suffering was defined as pain suffering—a component of affective pain (or emotional unpleasantness), which reflects both the physical threat of the pain and the long-term emotional meaning of the pain in one's life [27, 31]. The results showed that in IBS patients higher levels of anxiety and worry are associated with higher levels of suffering, which are related to limitations and perceived problems in performing physical activities due to physical health problems [27, 31].

According to a recent review [32], there are 10 instruments available for assessing suffering for both research and clinical purposes. Among all the examined instruments, Pictorial Representation of Illness and Self-Measure (PRISM) showed the best psychometric properties, such as construct validity and test-retest reliability, and exhibits ease of readability and comprehension, as well as sensitivity to change. Additionally, the PRISM-RII is very brief and easy to administer which, should it prove useful and informative for IBS, could be one of its advantages. The PRISM was designed as a generic measure of suffering and is not a typical questionnaire-type scale, but rather an abstract, pictorial measure. It yields two scores: one reflecting the subjective position of one's illness in relation to one's self (SIS: self-illness separation) and the other indicating the patient's perceived severity of the illness (IPM: illness perception measure) [28]. Previous studies indicate that PRISM does indeed measure suffering [33]. So far, it has been validated for use with a number of patient populations, including patients with systemic lupus, vitiligo, obesity, lung disease, psoriasis, chronic noncancer pain, diabetes, fertility problems, and breast cancer [32, 34]. To date however, PRISM has not been used for assessing suffering in IBS patients.

The aim of the present study was to evaluate the adequacy of PRISM as a measure of suffering in IBS patients. In other words, we wanted to examine its relationship with measures of QoL and anxiety, in order to determine if it could be useful for identifying patients with severe symptoms and/or severe QoL impairment, who would benefit from psychological interventions.

## 2. Methods

### 2.1. Participants

A total of 44 outpatients (32 female, 12 male) of the Gastroenterology Department, Clinical Hospital Centre Rijeka, participated in the study. Their age range was 21 to 69 years (*M* = 45.33, SD = 13.66); most of them were married or living with a partner (68.2%), employed (59.1%), and with a high school education (70.5%).

### 2.2. Questionnaires

#### 2.2.1. IBS-36 [35]

The IBS-36 measures health related QoL specific for IBS patients. It encompasses all areas of QoL relevant for IBS and has been shown to be sensitive enough to detect change after clinical interventions. It consists of 36 items, which participants rate on a scale of 0 to 6, indicating how often they experienced what is described in each item. A higher total score indicates a lower health related QoL [35].

#### 2.2.2. Medical Outcome Study Short-Form 36 [36]

The Medical Outcome Study Short-Form 36 (SF-36) is a measure of physical and mental health related QoL. It encompasses 4 domains in the area of physical health (physical functioning, role limitations due to physical health, bodily pain, and general health) and 4 domains in the area of mental health (role limitations due to emotional problems, energy/fatigue, emotional well-being, and social functioning). Two final scores are extracted: the physical component summary and the mental component summary, with higher scores reflecting better health related QoL [36].

#### 2.2.3. State-Trait Anxiety Inventory [37]

The STAI measures proneness or tendency toward anxiety. It consists of 20 items. The participants respond on a 4-point scale, marking how often they feel a certain way, in general. The final score is obtained by adding up responses for each item. A higher score indicates a higher proneness to anxiety [37].

#### 2.2.4. Visceral Sensitivity Index (VSI) [19]

The VSI is a measure of GI-specific anxiety. It consists of 15 items, referring to different aspects of fear and anxiety which may accompany appraisals of gastrointestinal sensations and discomfort. The participants' ratings on each item are added up to calculate the final score, with higher scores indicating higher levels of GI-specific anxiety [19].

#### 2.2.5. IBS Symptom Severity Scale

The IBS Symptom Severity Scale was used as a symptom diary. It was constructed based on the Gastrointestinal Symptom Diary [38], in order to measure patients' symptom severity. The scale contains 8 symptoms: constipation, diarrhoea, abdominal pain, abdominal tenderness, bloating, nausea, flatulence, and belching. Participants rated the severity of each symptom on a scale from 0 (absent) to 4 (debilitating) three times a day for two weeks. For each measurement time point, an average severity score was calculated by dividing the sum of all severities with the number of symptoms present (marked higher than 0). One final score for each participant was calculated as the 14-day average of symptom severity.

#### 2.2.6. Pictorial Representation of Illness and Self-Measure (PRISM-RII) [28]

The PRISM-RII (revised) is a visual measure of suffering. It consists of a large white circle (the patients' life), a small yellow circle (the patients' self), and three red circles (the patients' illness) of different sizes (one the size of the yellow circle, one smaller, and one bigger than the yellow disk). The participants' task is to choose one of the three red circles, which represents his/her illness most accurately and to place the red circle inside the white one (his/her life), which can be completely or partially on top of the yellow circle (his/her self). Since a paper version of this measure was used, the participants were asked to draw a circle (representing their illness) rather than just placing it inside the white circle (as in the original computer version of the measure). The PRISM-RII has two scores: self-illness separation (SIS), the distance between the centre of the yellow and red circles, in millimetres, and illness perception measure (IPM), ranging from 1 to 3, representing the three differently sized red circles (1 is the smallest). In this study, 31 participants chose the smallest red circle, 12 chose the middle-sized circle, and only one chose the larger red circle. For analysis purposes, that one participant was treated as part of the group that chose the medium sized circle. SIS reflects the subjective position of one's illness in relation to one's self, while IPM is an indicator of the perceived severity of the illness. A larger SIS (distance between the self and the illness representation) is considered to indicate a healthy adjustment to the illness [28].

Descriptive data for IBS-36, SF-36 (physical and mental component), STAI-T, VSI, SIS, and symptom severity obtained on this sample is presented in Table 1.

### 2.3. Procedure

The data was collected in the Clinical Hospital Centre in Rijeka, where all the participants were recruited. They completed the study in small groups of 2 to 7 participants. For each group, the procedure had three parts: the first and the third part of the study included completing a set of questionnaires (general information, STAI-T, and VSI in the first part; IBS-36, SF-36, and PRISM-RII in the third part) at the Clinical Hospital Centre, while the second part of the study lasted two weeks and was carried out individually by each participant during which time they kept a symptom diary three times a day: within two hours upon waking, between 16 and 18 hours in the afternoon, and within two hours before sleep. Participants were reminded about each measurement point via SMS.

## 3. Results

In order to examine the relationship between different measures of health related QoL and anxiety with measures of suffering (SIS and IPM), correlation analyses were performed.  Table 2 contains Pearson correlation coefficients for all the measures used.

Correlations between different measures of QoL are quite expected and similar to the results of previous studies [35]. IBS-36 is negatively related to both SF-36 scores (although correlation with the mental component does not reach a level of statistical significance; *p* = .053) and positively related to symptom severity. The physical and mental components of SF-36 correlate positively with each other. STAI-T is negatively correlated with both physical and mental component of SF-36, while VSI is positively related to IBS-36, STAI-T, and symptom severity and negatively with both SF-36 scores.

SIS negatively correlates with VSI, meaning that participants with lower visceral anxiety report a greater separation of self and illness. IPM correlates negatively with SIS and the mental component of SF-36 (for the physical component of SF-36 *p* = .07; for IBS-36 *p* = .08) and positively with VSI and symptom severity. Participants that perceive their illness as greater report a lower self-illness separation, a more impaired physical component of SF-36, and a higher visceral anxiety and symptom severity.

Considering that in this study the IPM measure only has two levels, small and medium size of illness, correlations might not be the most appropriate way of analysing its relationship with other measures of interest. Thus, we conducted* t*-tests, to determine whether participants who perceive their illness differently also differ on QoL and anxiety measures. Results are presented in Table 3.

As Table 3 shows, we found significant differences between participants who perceive their illness as small or medium, in SIS, symptom severity, and VSI, which is in line with the correlation analyses. Specifically, participants who perceive their illness as small represented their illness as more distant than their representation of self, showed a significantly lower average symptom severity, and had significantly lower GI-specific anxiety scores. Also in line with the correlation analyses, significant differences were found for the mental component of SF-36, but not for the physical component of SF-36. Participants who perceive their illness as small had a significantly higher mental component of SF-36, and while the same can be observed for the means of the physical component of SF-36, the difference did not reach a level of statistical significance (*p* = .07). All differences have medium effects sizes regardless of their statistical significance.

## 4. Discussion

This study was performed in order to examine the relationship between a relatively new measure of suffering and well-known measures of anxiety and QoL in IBS patients. The three QoL measures show moderately high significant intercorrelations (Table 2). IBS-36 is a specific QoL measure for IBS, containing items which refer to both problems with emotional and social functioning and items describing symptoms specific for IBS. Therefore, its relationship with the mental and physical components of SF-36 is quite expected and has been previously reported [35]. Symptom severity was the only outcome measure derived from the two-week diary data, and it shows a significant relationship only with IBS-36. In this case, symptom severity was the average intensity of symptoms reported in the two-week period. Since IBS-36 specifically measures dysfunction due to bowel difficulties, it was expected to correlate with the reported severity of symptoms in that period. On the other hand, it seems that QoL measured by SF-36 is too distal and general to be related to average reported symptom severity. Although there are findings linking pain severity to physical QoL measured by the SF-36 [7, 30], it seems that a greater number of symptoms, including abdominal distension, constipation, and diarrhoea, are related to IBS-specific QoL [30]. Also, some studies report that psychological states are more important for general QoL impairment in IBS patients than the severity of their symptoms [39].

Trait anxiety showed significant correlations with the mental and physical component of QoL, as well as with GI-related anxiety. Research consistently shows that, in IBS patients, anxiety levels are significantly related to QoL impairment [7, 11, 14, 30, 40]. In fact, psychological distress, including depression, negative affect, stress, and other anxiety-related states, has been related to lower QoL in IBS patients, which is the basis for using psychotherapy in alleviating symptoms and improving QoL, through the reduction of anxiety and depression [41]. GI-specific anxiety seems to be crucial for the concept of suffering in IBS patients. Our results show that it significantly correlates with all three measures of QoL, trait anxiety, symptom severity, and both PRISM-RII measures, self-illness separation and illness perception. Previous studies found that GI-specific anxiety is a good predictor of IBS diagnostic status and that it significantly predicts mental QoL [22], abdominal pain [42], and symptom severity [43], independently from anxiety and anxiety sensitivity [21]. GI-specific anxiety refers to feelings, thoughts, and behaviour arising from fear of GI symptoms. It includes fear and worry about GI symptoms, but also avoidance of the symptoms and situations in which they might appear [23]. Worry is defined as a relatively uncontrollable stream of thoughts and images with a negative valence, which represents an attempt of solving an issue with uncertain, but potentially negative outcomes [44]. Worry is ruminative and thus disruptive to problem-solving, promotes attentional vigilance and exaggerated threat-related beliefs, and can lead to negative emotional states. According to previous research [27], it also seems that, in IBS patients, worry is a significant predictor of suffering. Moreover, the effects of worry on suffering are mediated through catastrophizing, the tendency of exaggerating the threat value of pain [27]. In a study with chronic pain patients [45], SIS was negatively correlated with catastrophizing, more specifically with rumination, magnification, and helplessness. Considering the similarities between GI-specific anxiety, worry, and catastrophizing, the results of our study are in line with those findings. IBS patients, who worry about their symptoms more, are hypervigilant, and engage in avoidance behaviours, also perceive their illness as central in their lives or in other words experience more suffering. In fact, in this study, the SIS measure of suffering was related only to GI-specific anxiety.

Previous studies on suffering in other patient populations have found significant correlations between SIS and the physical and mental components of QoL [33, 45, 46], although that has not been the case for all QoL measures [28, 47]. Also, some of the correlations previously reported for SIS and IPM and SF-36 composite scores [46] are similar in magnitude to those obtained in this study; however due to our small sample size, ours failed to reach a level of statistical significance. Finally, there are studies which, in line with our results, reported no significant correlations between SIS and the physical and mental QoL [48]. Klis et al. [28] offer an explanation of such mixed results, proposing that low QoL is not necessarily experienced as suffering, unless the patient also lacks the ability to give it meaning, which could lead to inconsistent findings on correlations in different populations. Illness perception measure, the second PRISM measure, was successful in differentiating IBS patients based on the severity of their symptoms, mental QoL, GI-specific anxiety, and SIS. The two PRISM measures have a moderate negative correlation (*r* = −.35), which is in line with previous studies [28]. Even though patients only chose the small or the medium disk, those, who perceived their illness as more central to their self-concept, more often chose the bigger sized disk; that is, they consider their illness as more severe, compared to those who had a larger SIS. Patients, who perceived their illness as larger, had lower mental QoL, higher GI-specific anxiety, and more severe symptoms. IPM has previously been related to QoL measures in patients with diabetes [28], long-term cancer survivors [46], lung disease, psoriasis, fertility problems, breast cancer, and whiplash [34]. Moreover, a recent systematic review [49] on all available published data on PRISM reports that, in studies that used the revised PRISM task (PRISM-R and PRISM-RII), correlations with other variables, such as health status and well-being [34], were greater for IPM than SIS. Taking this into account, it would seem that our results are in line with studies using the revised PRISM task.

However, the presented results should be interpreted with caution, taking into account several limitations of the study. Unlike other medical conditions which were previously studied using the PRISM task, the diagnosis of IBS is based on a patient's subjective report about their symptoms, as is the case for other functional disorders. Also, the heterogeneous nature of IBS can pose a problem especially when dealing with samples of this size. The small sample size is the most significant limitation of this study, which could account for some differences in the obtained results compared to previously reported findings. Finally, no causal conclusions can be made, as the study is correlational in nature.

Keeping these limitations in mind, the presented results provide support for the usefulness of the PRISM task in IBS research and can offer possible directions for future studies on this topic. Anxiety-related states seem to be central for suffering in IBS patients, but additional information is needed on the relationship between suffering and cognitive and behavioural aspects of IBS, such as catastrophizing and avoidance behaviours. Using larger sample sizes would allow for modelling potential effects of those factors on suffering itself. This would be of significant value in understanding IBS and ultimately in relieving suffering in IBS patients.

## 5. Conclusion

Suffering is a state frequently reported by people who experience chronic pain, especially if the pain is uncontrollable and when its source is unknown. Since IBS is a chronic condition with characteristic abdominal pain or discomfort, whose cause is mainly unknown and episodes are unpredictable and uncontrollable, and based on the results of this study, it seems that suffering is an important topic for IBS which has received very little attention so far. Perceived severity of one's illness seems to be related to a number of factors, including symptom severity, emotional and social functioning limitations, and worry about GI symptoms. The use of PRISM-RII in the assessment of IBS patients could offer additional insight into their illness-related psychological functioning and help in discriminating patients with more prominent psychological difficulties.

## Figures and Tables

**Table 1:** Means, standard deviations, ranges, and reliability coefficients for IBS-36, SF-36 (physical and mental component), STAI-T, VSI, SIS, and symptom severity.

Scale	*M*	SD	Scale range	Obtained range	Cronbach alpha
IBS-36	50.43	32.52	0–216	0–141	.94
SF-36 PC	75.72	13.92	0–100	41.43–97.86	.82
SF-36 MC	66.92	19.36	0–100	21.07–95.36	.90
STAI-T	35.27	12.47	0–80	1–59	.92
VSI	27.84	14.14	0–60	2–55	.93
SIS	46.05	25.57	0–93	0–92	
Symptom severity	1.41	0.38	0–4	0.60–2.05	

**Table 2:** Correlations between health related QoL (IBS-36, physical and mental component of SF-36), anxiety (STAI-T and VSI), symptom severity, and suffering (SIS and IPM).

Scale	2.	3.	4.	5.	6.	7.	8.
1. IBS-36	−**.32**^**∗**^	−.29^†^	.27^†^	**.45** ^**∗∗**^	**.44** ^**∗∗**^	−.24	.27^†^
2. SF-36 PC		**.50** ^**∗∗**^	**−.48** ^**∗∗**^	**−.38** ^**∗**^	−.29^†^	.16	−.27^†^
3. SF-36 MC			**−.48** ^**∗∗**^	**−.34** ^**∗**^	−.17	.07	**−.33** ^**∗**^
4. STAI-T				**.47** ^**∗∗**^	.18	−.22	.28^†^
5. VSI					**.31** ^**∗**^	**−.37** ^**∗**^	**.34** ^**∗**^
6. Symptom severity						−.20	**.37** ^**∗**^
7. SIS							**−.35** ^**∗**^
8. IPM							—

^†^ *p* < .10; ^*∗*^*p* < .05; ^*∗∗*^*p* < .01.

**Table 3:** Differences in health related QoL (IBS-36, physical and mental component of SF-36), anxiety (STAI-T and VSI), symptom severity, and self-illness separation (SIS) between patients with low and medium IPM score.

	*t*	Cohen's *d*	*M*_low_ (SD)	*M*_medium_ (SD)
IBS-36	−1.78^†^	0.43	44.90 (31.52)	63.62 (32.24)
SF-PC	1.83^†^	0.60	78.14 (13.28)	69.95 (14.23)
SF-MC	**2.24** ^**∗**^	**0.70**	**70.98 (17.03)**	**57.25 (21.78)**
STAI-T	−1.86^†^	0.66	33.06 (13.13)	40.54 (9.15)
VSI	**−2.36** ^**∗**^	**0.83**	**24.74 (14.49)**	**35.23 (10.38)**
Symptom severity	**−2.54** ^**∗**^	**0.76**	**1.32 (0.38)**	**1.62 (0.29)**
SIS	**2.44** ^**∗**^	**0.80**	**51.81 (24.00)**	**32.31 (24.76)**

^†^ *p* < .10; ^*∗*^*p* < .05.

## References

[1] Canavan C., West J., Card T. (2014). The epidemiology of irritable bowel syndrome. *Clinical Epidemiology*.

[2] Longstreth G. F., Thompson W. G., Chey W. D., Houghton L. A., Mearin F., Spiller R. C., Drossman D. A., Corazziari E., Delvaux M. (2006). Functional bowel disorders. *Rome III: The Functional Gastrointestinal Disorders*.

[3] El-Salhy M. (2012). Irritable bowel syndrome: diagnosis and pathogenesis. *World Journal of Gastroenterology*.

[4] Deary V., Chalder T., Sharpe M. (2007). The cognitive behavioural model of medically unexplained symptoms: a theoretical and empirical review. *Clinical Psychology Review*.

[5] Hauser G., Pletikosic S., Tkalcic M. (2014). Cognitive behavioral approach to understanding irritable bowel syndrome. *World Journal of Gastroenterology*.

[6] Ten Berg M. J., Goettsch W. G., Van Den Boom G., Smout A. J. P. M., Herings R. M. C. (2006). Quality of life of patients with irritable bowel syndrome is low compared to others with chronic diseases. *European Journal of Gastroenterology and Hepatology*.

[7] Rey E., García-Alonso M. O., Moreno-Ortega M., Alvarez-Sanchez A., Diaz-Rubio M. (2008). Determinants of quality of life in irritable bowel syndrome. *Journal of Clinical Gastroenterology*.

[8] Wong R. K. M., Drossman D. A. (2010). Quality of life measures in irritable bowel syndrome. *Expert Review of Gastroenterology and Hepatology*.

[9] Huang W.-W., Zhou F.-S., Bushnell D. M., Diakite C., Yang X.-H. (2007). Cultural adaptation and application of the IBS-QOL in China: a disease-specific quality-of-life questionnaire. *Quality of Life Research*.

[10] Naliboff B. D., Kim S. E., Bolus R., Bernstein C. N., Mayer E. A., Chang L. (2012). Gastrointestinal and psychological mediators of health-related quality of life in IBS and IBD: a structural equation modeling analysis. *American Journal of Gastroenterology*.

[11] Tkalčić M., Hauser G., Štimac D. (2010). Differences in the health-related quality of life, affective status, and personality between irritable bowel syndrome and inflammatory bowel disease patients. *European Journal of Gastroenterology and Hepatology*.

[12] Cain K. C., Headstrom P., Jarrett M. E. (2006). Abdominal pain impacts quality of life in women with irritable bowel syndrome. *The American Journal of Gastroenterology*.

[13] Sperber A. D., Carmel S., Atzmon Y. (2006). Use of the functional bowel disorder severity index (FBDSI) in a study of patients with the irritable bowel syndrome and fibromyalgia. *American Journal of Gastroenterology*.

[14] Cho H. S., Park J. M., Lim C. H. (2011). Anxiety, depression and quality of life in patients with irritable bowel syndrome. *Gut and Liver*.

[15] Quigley E. M. M. (2005). Irritable bowel syndrome and inflammatory bowel disease: interrelated diseases?. *Chinese Journal of Digestive Diseases*.

[16] Sugaya N., Nomura S. (2008). Relationship between cognitive appraisals of symptoms and negative mood for subtypes of irritable bowel syndrome. *BioPsychoSocial Medicine*.

[17] Creed F. H., Levy R., Bradley L., Drossman D. A., Corazziari E., Delvaux M. (2006). Psychosocial aspects of functional gastrointestinal disorders. *Rome III: The Functional Gastrointestinal Disorders*.

[18] Hood S. D., Shufflebotham J. Q., Hendry J. (2008). Irritable bowel syndrome patients exhibit depressive and anxiety scores in the subsyndromal range. *The Open Psychiatry Journal*.

[19] Labus J. S., Bolus R., Chang L. (2004). The visceral sensitivity index: development and validation of a gastrointestinal symptom-specific anxiety scale. *Alimentary Pharmacology and Therapeutics*.

[20] Hazlett-Stevens H., Craske M. G., Mayer E. A., Chang L., Naliboff B. D. (2003). Prevalence of irritable bowel syndrome among university students: the roles of worry, neuroticism, anxiety sensitivity and visceral anxiety. *Journal of Psychosomatic Research*.

[21] Saigo T., Tayama J., Hamaguchi T. (2014). Gastrointestinal specific anxiety in irritable bowel syndrome: validation of the Japanese version of the visceral sensitivity index for university students. *BioPsychoSocial Medicine*.

[22] Jerndal P., Ringström G., Agerforz P. (2010). Gastrointestinal-specific anxiety: an important factor for severity of GI symptoms and quality of life in IBS. *Neurogastroenterology and Motility*.

[23] Labus J. S., Mayer E. A., Chang L., Bolus R., Naliboff B. D. (2007). The central role of gastrointestinal-specific anxiety in irritable bowel syndrome: further validation of the visceral sensitivity index. *Psychosomatic Medicine*.

[24] Cassell E. J. (1982). The nature of suffering and the goals of medicine. *New England Journal of Medicine*.

[25] Chapman C. R., Gavrin J. (1999). Suffering: the contributions of persistent pain. *The Lancet*.

[26] Krikorian A., Limonero J. T. (2012). An integrated view of suffering in palliative care. *Journal of Palliative Care*.

[27] Lackner J. M., Quigley B. M. (2005). Pain catastrophizing mediates the relationship between worry and pain suffering in patients with irritable bowel syndrome. *Behaviour Research and Therapy*.

[28] Klis S., Vingerhoets A. J. J. M., de Wit M., Zandbelt N., Snoek F. J. (2008). Pictorial representation of illness and self measure revised II (PRISM-RII)—a novel method to assess perceived burden of illness in diabetes patients. *Health and Quality of Life Outcomes*.

[29] Lackner J. M., Quigley B. M., Blanchard E. B. (2004). Depression and abdominal pain in IBS patients: the mediating role of catastrophizing. *Psychosomatic Medicine*.

[30] Lee V., Guthrie E., Robinson A. (2008). Functional bowel disorders in primary care: factors associated with health-related quality of life and doctor consultation. *Journal of Psychosomatic Research*.

[31] Lackner J. M., Jaccard J., Blanchard E. B. (2005). Testing the sequential model of pain processing in irritable bowel syndrome: a structural equation modeling analysis. *European Journal of Pain*.

[32] Krikorian A., Limonero J. T., Corey M. T. (2013). Suffering assessment: a review of available instruments for use in palliative care. *Journal of Palliative Medicine*.

[33] Büchi S., Buddeberg C., Klaghofer R. (2002). Preliminary validation of PRISM (pictorial representation of illness and self measure)—a brief method to assess suffering. *Psychotherapy and Psychosomatics*.

[34] Wouters E., Reimus J., Van Nunen A., Blokhorst M., Vingerhoets A. (2008). Suffering quantified? feasibility and psychometric characteristics of 2 revised versions of the pictorial representation of illness and self measure (PRISM). *Behavioral Medicine*.

[35] Groll D., Vanner S. J., Depew W. T. (2002). The IBS-36: a new quality of life measure for irritable bowel syndrome. *American Journal of Gastroenterology*.

[36] Ware J. E., Sherbourne C. D. (1992). The MOS 36-item short-form health survey (SF-36): I. conceptual framework and item selection. *Medical Care*.

[37] Spielberger C. D. (2000). *Priručnik za Upitnik anksioznosti kao stanja i osobine ličnost [Manual for the State-Trait Anxiety Inventory]*.

[38] Blanchard E. B. (2001). *Irritable Bowel Syndrome: Psychosocial Assessment and Treatment*.

[39] Michalsen V. L., Vandvik P. O., Farup P. G. (2015). Predictors of health-related quality of life in patients with irritable bowel syndrome. A cross-sectional study in Norway. *Health and Quality of Life Outcomes*.

[40] Spiegel B. M. R., Gralnek I. M., Bolus R. (2004). Clinical determinants of health-related quality of life in patients with irritable bowel syndrome. *Archives of Internal Medicine*.

[41] Jamali R., Jamali A., Poorrahnama M. (2012). Evaluation of health related quality of life in irritable bowel syndrome patients. *Health and Quality of Life Outcomes*.

[42] Patel A., Cassell B., Kumar M., Gyawali C. P., Sayuk G. S. (2012). 35 disturbed sleep worsens ibs pain symptoms: an effect of gastrointestinal (GI) specific anxiety?. *DDW Abstract Supplement to Gastroenterology*.

[43] McKinnon A. C., Van Oudenhove L., Tack J., Jones M. (2015). The association of personality, appraisal, catastrophising and vigilance with gastrointestinal symptom-specific anxiety. *Journal of Health Psychology*.

[44] Borkovec T. D., Robinson E., Pruzinsky T., DePree J. A. (1983). Preliminary exploration of worry: some characteristics and processes. *Behaviour Research and Therapy*.

[45] Kassardjian C. D., Gardner-Nix J., Dupak K., Barbati J., Lam-McCullock J. (2008). Validating PRISM (pictorial representation of illness and self-measure) as a measure of suffering in chronic non-cancer pain patients. *Journal of Pain*.

[46] Lehmann V., Oerlemans S., Van De Poll-Franse L. V., Vingerhoets A. J. J. M., Mols F. (2011). Suffering in long-term cancer survivors: an evaluation of the PRISM-R2 in a population-based cohort. *Quality of Life Research*.

[47] Reimus J. L. M., Vingerhoets A. J. J. M., Soons P. H. G. M., Korstanje M. J. (2007). Suffering in psoriasis patients: its relation with illness severity and subjective well-being. *International Journal of Dermatology*.

[48] Denton F., Sharpe L., Schrieber L. (2004). PRISM: enmeshment of illness and self-schema. *Psychotherapy and Psychosomatics*.

[49] Sensky T., Büchi S. (2016). PRISM, a novel visual metaphor measuring personally salient appraisals, attitudes and decision-making: qualitative evidence synthesis. *PLoS ONE*.

